# Exploring the relationship between dystonia and STN-DBS in Parkinson’s disease: insights from a single-centre cohort

**DOI:** 10.1007/s10072-025-08230-7

**Published:** 2025-05-15

**Authors:** Luigi G. Remore, Delia Gagliardi, Linda Borellini, Alfonso Fasano, Valeria Lo Faso, Filippo Cogiamanian, Enrico Mailand, Gloria Valcamonica, Elena Pirola, Luigi Schisano, Antonella M. Ampollini, Giulio A. Bertani, Giorgio Fiore, Antonio D’Ammando, Leonardo Tariciotti, Giovanni Marfia, Stefania Elena Navone, Sergio Barbieri, Marco Locatelli

**Affiliations:** 1 Neurosurgery Unit, Fondazione IRCCS Ca’ Granda Ospedale Maggiore Policlinico, Milan, Italy; 2 Department of Pathophysiology and Transplantation, University of Milan, Milan, Italy; 3 Neuropathophysiology Unit, Fondazione IRCCS Ca’Granda Ospedale Maggiore Policlinico, Milan, Italy; 4 Edmond J. Safra Program in Parkinson’s Disease, Morton and Gloria Shulman Movement Disorders Clinic, Toronto Western Hospital, University Health Network, Toronto, ON Canada; 5 Division of Neurology, University of Toronto, Toronto, ON Canada; 6 Krembil Brain Institute, Neuroscience, Toronto, ON Canada; 7 Laboratory of Experimental Neurosurgery and Cell Therapy, Unit of Neurosurgery, Fondazione IRCCS Ca’ Granda Ospedale Maggiore Policlinico, Milan, Italy

**Keywords:** Deep brain stimulation, Parkinson’s disease, Dystonia, Movement disorders, Side effects

## Abstract

**Introduction:**

Motor side effects may emerge after deep brain stimulation (DBS) of the subthalamic nucleus (STN) in Parkinson’s disease (PD) patients. Out of 60 PD patients, we observed 16 patients displaying de novo dystonic symptoms after the implantation and 11 dystonic PD patients without benefit from the stimulation. We hypothesized that a common neural pathway may cause dystonia in both conditions. Our study aims to investigate the clinical and connectivity substrates of dystonia after STN-DBS.

**Methods:**

We divided our cohort into four groups: 16 patients displaying dystonia after STN-DBS, 11 patients with previously known dystonia not improving after surgery, 14 patients with dystonic symptoms relieved by the stimulation and 19 controls who never experienced dystonia. MANOVA was used to compare clinical data and the distance of the active contact center from the STN border among the four groups. Finally, we reconstructed the “sour” spots for dystonic symptoms and the associated structural and functional connectivity using a Parkinsonian normative connectome.

**Results:**

De novo dystonic and not-improved dystonic patients had a statistically significant longer PD duration before surgery (p = 0.001) and a greater active contact-STN distance (p < 0.001). Moreover, the “sour” spots were similar in both groups and structural and functional connectivity profiles were associated with brain areas correlated with dystonia pathophysiology (cerebellum, midbrain, parietal and temporal cortices).

**Conclusions:**

We formulated a two-hit model for dystonia after STN-DBS: a clinical feature of Parkinsonian patients causes predisposing altered plasticity contributing to dystonic symptoms development when coupled with the stimulation of dystonia-related subcortical and cortical structures.

**Supplementary Information:**

The online version contains supplementary material available at 10.1007/s10072-025-08230-7.

## Introduction

Deep brain stimulation (DBS) targeting the subthalamic nucleus (STN) has been performed for decades for the treatment of Parkinson’s disease (PD) and its efficacy is worldwide acknowledged nowadays [1]. STN-DBS is indicated for PD patients in whom longstanding L-dopa therapy starts causing disabling motor side effects related to a non-steady oscillation of the drug’s concentration in the blood. These manifest as hyperkinetic ON-phenomena (dyskinetic movements during high L-dopa blood concentration) and hypokinetic OFF-phenomena (enhanced bradykinesia during low levodopa blood concentration), even though mixed-type effects with respect to levodopa dose are possible [2].

Motor side effects caused by STN stimulation have been known since the earliest experience with DBS [3]. Most of the stimulation-related side effects are tested intraoperatively during surgery and are usually responsive to changes in stimulation parameters: contralateral muscle contractions due to laterally spreading of the stimulation to cortico-spinal fibers, contralateral gaze deviation due to stimulation of descending fibers from the frontal eye field and ipsilateral diplopia and eye deviation due to medial activation of the third cranial nerve. Nonetheless, chronic stimulation-related motor side effects have also been described and their treatment has been reported to be more troublesome. Among these, dyskinesias is the most common and usually manifests as contralateral hemiballismus or hemichorea and can arise shortly after electrode implantation or within the first three months thereafter [4]. While dyskinesias may be due to micro-lesional effects in early cases, direct STN overstimulation is considered the cause of delayed ones. Most of the stimulation-related dyskinesias improve by reducing the voltage delivered to STN or by using more dorsal contacts in order to stimulate the caudal Zona Incerta (cZI), which is supposed to provide a robust anti-dyskinetic effect [5]. However, anecdotal cases of treatment-unresponsive dyskinesia have been reported, even causing life-threatening complications [6] or necessitating conversion to DBS of the globus pallidus pars interna (GPi) [7]. Apraxia of eyelid opening (AEO) is a rarer chronic stimulation-related motor side effect (incidence variably reported between 2 and 31%) and is considered a form of focal dystonia [8]. AEO is thought to emerge from the laterally spreading of currents toward the corticobulbar/corticospinal fibers and its treatment approach ranges from increasing the levodopa dose to changing stimulation parameters (lower voltages and higher frequencies) or botulinum toxin injection for the most refractory cases [9].

Dystonia and PD are closely related movement disorders and they share some pathophysiological mechanisms. Dystonia can coexist in up to 30% of PD patients and it may also precede overt parkinsonian symptoms in some cases [10]. Moreover, GPi-DBS for the treatment of dystonia may cause the worsening of pre-existing dystonia and even parkinsonism [11].

In the present study, we retrospectively analyzed a cohort of 60 PD patients who underwent STN-DBS in our center. Among these patients, some displayed purely dystonic symptoms resembling sporadic forms of dystonia and persisting several months after surgery, while others had dystonic symptoms before STN-DBS and did not respond to stimulation despite several adjustments of stimulation parameters.

Our hypothesis is that a common neural pathway may be responsible for both de novo dystonic symptoms and the unresponsiveness of pre-existent dystonic symptoms to stimulation.

To prove our hypothesis, we compared patients’ clinical characteristics and active contact location relative to STN and generated heat maps focusing on the sour-spots, defined as the sites of stimulation associated with a side effect, as opposed to sweet-spot, i.e. the sites of stimulation associated with improvement [12]. Finally, we reconstructed the structural and functional connectivity profiles or fingerprints related to the dystonic side effect.

## Materials and methods

### Patients’ selection and group comparison

We retrospectively reviewed the clinical database of our institution and searched for PD patients who underwent bilateral STN-DBS between 2014 and 2019. 60 patients resulted from our search. The selected patients were divided into four groups: 16 patients displaying de novo dystonic symptoms after the implantation, 11 patients with pre-existing dystonia who did not display symptoms improvement at follow-up clinical evaluations, 14 pre-dystonic patients who markedly improved after turning on the implant and 19 control patients without dystonic symptoms both before and after surgery.

The following variables were compared among the four groups: demographic data (sex, age at PD onset and implantation), clinical data (disease duration, the first lateralized side affected by PD, LEDD [levodopa equivalent daily dose] calculated before the implantation and ΔLEDD, i.e. the difference between calculated LEDD pre- and 1-year post-implantation), clinical scores before and one year after the surgery (MDS-UPDRS III, MDS-UPDRS IV, MDS-NMS, PDQ39) and active contact distance from STN. Moreover, additional clinical variables were collected for dystonic patients: body side with dystonic symptoms, concordance between the side affected by PD and the side affected by dystonia, type of dystonia (focal, multifocal, hemi-dystonia, segmental, generalized) and moment of dystonic symptoms occurrence in relation to levodopa peak dose (ON, OFF, persistent).

All PD patients selected for this study were followed by two expert attending neurologists (F.C. and L.B.) in the Movement Disorders Clinic and all surgical procedures were performed by the same attendant neurosurgeons (M.L. and E.P.) at the Neurosurgery Department of our institution. Ethical review and approval were waived for this retrospective study following local legislation and institutional requirements. All patients gave informed written consent in accordance with the Declaration of Helsinki.

### Surgical procedure

Our surgical technique consisted of a two-step procedure as previously described [13, 14]. Briefly, the first step was conducted under local anaesthesia in the awake patient. Before surgery, a Cosman-Roberts-Wells stereotactic frame (Radionics by Integra, Plainsboro, New Jersey, USA) was placed on the patient’s head and a stereotactic CT scan was performed. A dedicated workstation (Istereotaxy by Brainlab, Kapellenstrat, Germany) was used to co-register the CT scan with the MRI performed the day before to plan STN targeting. Recording microelectrodes (microTargeting Electrode™, FHC Inc, Bowdoin, Maine, USA) were inserted before lead implantation and the electrophysiologic activity of STN was tested. A neurologist checked for possible side effects during intraoperative stimulation. After the correct electrophysiologic localization of the nucleus, DBS leads (Model 3389 by Medtronic Inc., Minneapolis, Minnesota, USA) were implanted bilaterally. A second surgery step was scheduled within seven days after the first one and performed under general anaesthesia. During this surgical time, intracranial electrodes were elongated with lead extenders, which were then connected to an IPG Medtronic Activa PC implanted into a subcutaneous pouch in the right hemithorax. Patients were subsequently discharged and the stimulation was turned on three to six weeks after surgery during a second hospitalization.

### Leads reconstructions and active contact localization

DBS electrodes were localized using the advanced processing pipeline of Lead-DBS Ver 2.6, as described in previous studies [15]. Briefly, postoperative CT was linearly co-registered to pre-operative MRI (T1-weighted and T2-weighted sequences) and then non-linearly co-registered to the MNI 2009b asymmetric space by using the advanced normalization tools ANTs. All registration steps were inspected and refined when appropriate. Brain shift correction was applied as implemented in Lead-DBS. DBS contacts were automatically pre-reconstructed using the phantom-validated and fully automated PaCER method and manually refined when appropriate. Atlas segmentations in this manuscript are defined by the DISTAL atlas to visualize STN [16].

With the Lead-Group tool in Lead-DBS [15], group visualization of the reconstructed electrodes was performed and the millimeter distance from the center of gravity of the active contact to the nearest voxel center of STN was calculated.

### Sour-spot estimation and structural and functional fingerprint generation

VTAs (volumes of tissue activated) were computed for each patient using the stimulation parameters (active contacts and amplitude in volts) as recorded at the last follow-up visit. VTAs were estimated using the finite element method (FEM)-based FieldTrip SimBio pipeline in Lead-DBS [17]. As reported by previous studies [12], VTAs were modeled assuming a homogenous tissue conductivity of 0.1 S/m and thresholded to binary three-dimensional volumes by applying an electric field cutoff of 0.2 V/mm to approximate the gold standard of axon cable models. An N-image approach [18] was chosen to generate the sour-spot for de novo dystonic patients and PD patients with dystonia that had not improved after implantation by employing the Sweetspot Explorer tool in Lead-DBS. The sour spots were then wrapped in the MNI space and superimposed on the Distal atlas to visualize their relationship with STN, with the white matter tracts related to clinical outcomes in GPi-DBS for dystonia on the Dystonia Response Tract Atlas [19] and with the ZI on the Zona Incerta Atlas [20]

Connectivity profiles or fingerprints for the sour spots and the mean VTAs were generated by employing the Lead Connectome Mapper tool in Lead-DBS. The sour-spots were used as seeds for both structural and functional connectomic mapping within a normative Parkinsonian connectome formed by 85 PD patients from the Parkinson’s Progression Markers Initiative (PPMI; www.ppmi-info.org) database [21]. This preprocessed normative connectome has been used in previous studies and processing details are reported elsewhere [17, 21].

### Statistical analysis

Descriptive statistics, frequencies and percentages were used to report demographic characteristics. A Shapiro–Wilk normality test was used to assess normality across selected variables along with a close inspection of the data plotted on histograms and Q-Q graphs. Continuous variables were reported as means plus standard deviations. Categorical variables were reported as absolute numbers and percentages. MANOVA was employed to perform multivariate comparisons among the four groups of patients. In this context, the Pillai-Bartlett trace (V) multivariate test was used as a test for overall statistical significance among all variables and ANOVA univariate F-statistic as a follow-up test in case of multivariate significance. Finally, two posthoc tests were used to uncover specific differences in the statistically significant variables among the four groups: Bonferroni if Levene's test of equality of error variances was not violated and Games-Howell in the case of Levene’s test violation. Variables specific to dystonic symptoms were categorical and the Chi-Square test was used to compare them among the three groups of dystonic PD patients (de novo dystonic, improved pre-dystonic and not-improved pre-dystonic). For all traditional hypotheses, p values < 0.05 were considered statistically significant. Statistical analysis was performed using SPSS (version 28.0, IBM).

## Results

### Whole cohort demographics

Most patients were males (58.3%) with a mean age at implantation of 61.75 ± 8.72 years. The mean age at PD onset was 46.62 ± 7.92 years, the mean disease duration before DBS was 15 ± 5.02 years and the body side first affected by PD was the left one in 56.7% of cases. The calculated mean LEDD before surgery and ΔLEDD were 1111.57 ± 335.05 mg/day and 606.83 ± 242.54 mg/day, respectively. Finally, the mean distance of the active contact from STN was 0.5 ± 0.7 mm for the left side and 0.63 ± 0.6 for the right side.

### Comparisons among the four groups

The multivariate test was statistically significant (V = 1.08, F(24,132) = 1.65, p = 0.015) and the follow-up univariate analysis confirmed significant differences among the four groups for three variables (Table 1): the distance of the active contact from STN bilaterally and the duration of the disease before the implantation.

**Table 1:** Clinical variables for the four groups of patients

Variable	De novo dystonia	No dystonia	Pre-dystonia not improved	Pre-dystonia improved	F^7^	p-value
AC-STN^1^ Left	0.91 ± 1.1	0.154 ± 0.12	0.82 ± 0.5	0.2 ± 0.3	6.44	0.001^*^
AC-STN^1^ Right	1.06 ± 0.7	0.266 ± 0.26	1.11 ± 0.305	0.273 ± 0.371	16.73	< 0.001*
Age at implantation^2^	62.25 ± 7.7	61.05 ± 9.33	62.36 ± 10.3	61.57 ± 8.6	0.074	0.974
Age at Onset^3^	45.63 ± 8.26	47.37 ± 8	44.55 ± 5.33	47.21 ± 9.35	0.388	0.762
Disease duration^4^	16.63 ± 5	12.21 ± 3.42	18.64 ± 6.56	13.64 ± 2.8	5.977	0.001^*^
LEDD pre^5^	1225 ± 390.6	1150 ± 294.3	944.45 ± 362	1064.8 ± 268	1.75	0.167
ΔLEDD^6^	645.25 ± 260	640 ± 217.18	487 ± 250	605 ± 227	1.2	0.321
MDS- UPDRS III pre^8^	38.81 ± 12.46	38.85 ± 12.1	41 ± 2.5	44.8 ± 10.25	1.042	0.381
MDS- UPDRS III post	22.18 ± 8.55	23.47 ± 9.23	20 ± 4.65	24.35 ± 8.53	0.7	0.564
MDS- UPDRS IV pre^9^	9.81 ± 3.44	8.8 ± 2.15	8.6 ± 0.8	8.8 ± 0.42	0.93	0.434
MDS- UPDRS IV post	2.18 ± 0.4	2.42 ± 0.2	2.3 ± 0.46	2.07 ± 0.26	0.254	0.86
MDS-NMS pre^10^	48.12 ± 20.66	42.42 ± 20.3	45.72 ± 3.2	45.57 ± 2.8	0.383	0.766
MDS-NMS post	30.37 ± 4.06	31 ± 12.6	32 ± 0.4	29.42 ± 5	0.215	0.886
PDQ39 pre^11^	57.56 ± 27.61	53 ± 23	55.5 ± 5.85	54.45 ± 0.8	0.162	0.922
PDQ39 post	30.68 ± 0.47	31.36 ± 14.7	30.81 ± 0.4	30.85 ± 0.36	0.022	0.995

1: distance of the center of the active contact (AC) from the subthalamic nucleus (STN) in mm; 2: mean age in years at the time of implantation; 3: mean age in years at the time of Parkinsons’s disease diagnosis; 4: mean duration of Parkinson’s disease in years until the surgery; 5: levodopa equivalent daily dose before the implantation in mg/day; 6: the difference between calculated LEDD pre- and 1-year post-implantation; 7: F-statistics and p-value reported from follow-up univariate comparison among the clinical variables of the four groups by using ANOVA; 8: Movement Disorder Society-Unified Parkinson’s Disease Rating Scale part III before and after the surgery; 9: Movement Disorder Society-Unified Parkinson’s Disease Rating Scale part IV before and after the surgery; 10: Movement Disorder Society-Non-Motor Rating Scale before and after the surgery; 11: The Parkinson’s Disease Questionnaire 39 Items before and after the surgery. ^*^p < 0.005 was considered statistically significant

The post-hoc test (Supplementary Table 1) revealed that the distance of the active contact from STN was significantly greater in de novo dystonic patients than non-dystonic for both the left (p = 0.004) and the right (p = 0.002) side. Similarly, the patients with dystonic symptoms who did not improve after DBS had a greater distance from STN in the left (p = 0.01) and right (p < 0.001) active contact. Furthermore, de novo dystonic had a statistically significant longer (p = 0.032) disease duration than the non-dystonic, such as the not-improved patients who suffered from PD for a longer time than improved patients (p = 0.04).

No statistically significant gender differences were found among the four groups (p = 0.536) (Supplementary Fig. 1).

### Clinical characteristics of dystonia

Of the 41 patients presenting with dystonia either before or after DBS, dystonic symptoms were left-sided in 56.1% of patients and concordant with the first hemibody affected by PD symptoms in 65.9% of cases. Dystonic postures were more frequently manifested in the OFF-levodopa state (68.3%), while only a minority of patients had dystonia in ON (24.4%) or displayed persistent dystonia (7.3%). Moreover, the type of dystonic symptoms was distributed among the patients as follows: 41.5% focal, 26.8% segmental, 2.4% multifocal, 17.1% hemidystonia and 12.2% generalized. Detailed characterization of body segments affected in each patient is described in Table 2. No patient had AEO after DBS.

**Table 2:** Description of body segments involved in each patient with dystonia

Dystonia Type	Number of patients
Focal	17
Foot inversion	6
Hand gripping	2
Wrist flexion	1
Cervical	5
Axial	3
Segmental	11
Cervical and upper limb torsion	2
Leg and foot torsion	7
Axial and lower limb torsion	2
Multifocal	1
Four limbs torsion	1
Hemidystonia	7
Generalized	5
Axial, harm and wrist torsion, leg and foot inversion	1
Axial, cervical, blepharospasm, foot inversion	1
Axial, wrist and hand torsion, foot inversion	1
Axial, cervical, foot inversion	1
Axial, foot inversion	1

No dystonic symptom improved with levodopa dose modification. Specifically, patients were convocated for an L-Dopa challenge test in the outpatient clinic after overnight withdrawal of antiparkinsonian medications. During the test, an attending neurologist conducted a full neurological examination and dystonic symptoms were evaluated according to the objective visit and the subjective improvement referred by the patients and the caregivers.Worsening of dystonia at levodopa peak dose was experienced by 60% of pre-dystonic patients (15/25) and 62.5% of de novo dystonic patients (10/16). No statistically significant differences (p = 0.41) resulted when comparing the detrimental effect of levodopa between patients based on the dystonia response to STN-DBS (de novo dystonic, pre-dystonic improved, pre-dystonic not-improved). Nevertheless, dystonic symptoms improved in 9 out of 16 de novo dystonic patients (56.2%) after changes in programming parameters. Specifically, dystonic postures subdue by reducing the intensity of the stimulation for some patients. However, other patients displayed poor tremor or bradykinesia control when lowering the stimulation threshold below 1.5–2 V. Therefore, interleaving programming was set for this latter patient group by activating the more dorsal contact at 1.1–1.5 V.

No statistically significant differences were found in any dystonic clinical characteristics among the three groups of dystonic patients (Supplementary Fig. 2).

### Sour-spot exploration

**Fig. 1:**
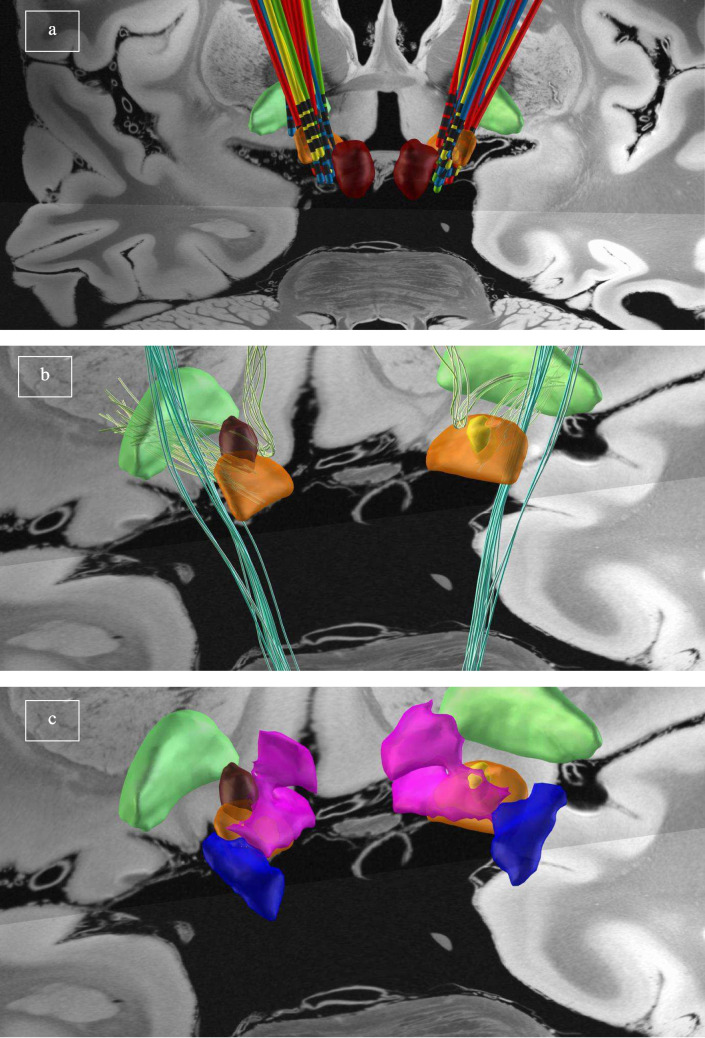
Group-level reconstruction of patients’ electrodes and sour-spot exploration. All reconstructions are rendered in Lead-DBS 3D viewer with the 7 T MNI 2009b asymmetric T1 as background. Subcortical structures are identified according to DISTAL Atlas: subthalamic nucleus (orange), red nucleus (red), globus pallidum pars interna (green). *Panel ***a***:* patients’ electrodes reconstruction at group level and their position relative to the subthalamic nucleus. Electrodes are color-coded according to the dystonia status: control patients are colored in red, de novo dystonic patients in yellow, improved pre-dystonic patients in blue, not-improved pre-dystonic patients in green. *Panel ***b***:* No differences were found between the left and right sides in the two sour-spot. On the left, the sour-spot for de novo dystonic patients is colored in brown; on the right, the sour-spot for pre-dystonic patients without benefit from DBS is colored in yellow. Both sour-spots extend cranially from the STN surface, but the former has a more pronounced anterolateral extension in close contact with fibers negatively associated with clinical outcome in GPi-DBS for cervical dystonia (fasciculus lenticularis, in light green) as described by [19]. Note that the fibers negatively associated with clinical outcome in GPi-DBS for generalized dystonia (striatopallidofugal tracts of the posterior comb system) are located more dorsally than the two sour-spots. *Panel ***c***:* Sour-spot’s relation with the subcortical structures as depicted by the Zona Incerta Atlas [20]. Both sour-spots get in touch medially with the rostral zona incerta (pink). Note that the caudal zona incerta (blue) resides dorsal to STN

3D exploration of the two generated sour-spots (Fig. 1) highlighted that both extended cranially from the STN border. Additionally, the sour spots of the de novo dystonic patients had a significant anterolateral extension towards fibers negatively associated with clinical outcome in GPi-DBS for cervical dystonia (namely fibers from the fasciculus lenticularis) as described by Horn et al. [19]. Moreover, the medial components of the two sour-spots were in close contact with the rostral zona incerta (rZI) as represented in the atlas by Lau et al. [20].

### Structural and functional fingerprints

Structural and functional fingerprints were almost identical for de-novo dystonic patients and non-improved pre-dystonic patients.

**Fig. 2:**
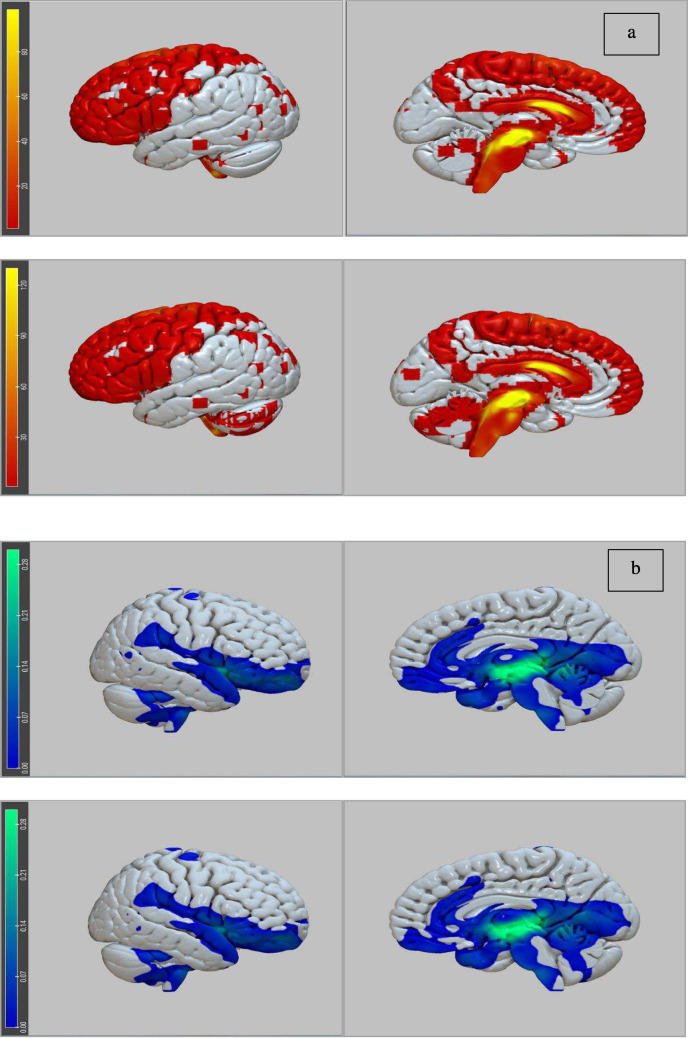
Structural and functional fingerprint of the two sour-spots. *Panel ***a***:* Structural connectivity profile of the lateral and medial surfaces of the left hemisphere for the sour-spot of de novo dystonic patients *(upper row)* and not-improved pre-dystonic patients *(lower row)*. The structural fingerprint of the two sour-spots shows prevalent connections with the brainstem, cerebellum, thalamus, motor and premotor areas, supplementary e pre-supplementary areas, part of the precuneus, the upper part of the primary somatosensory and the posterior parietal areas. *Panel ***b***:* Functional connectivity profile of the lateral and medial surfaces of the right hemisphere for the sour-spot of de novo dystonic patients *(upper row)* and not-improved pre-dystonic patients *(lower row)*

The structural connectivity profile showed prevalent connections with the brainstem, cerebellum, thalamus, motor and premotor areas, supplementary and pre-supplementary areas, part of the precuneus and the upper part of the primary somatosensory and the posterior parietal areas (Fig. 2a). The functional connectivity map revealed positive correlations with the anterior pons and midbrain, cerebellum, anterior cingulate cortex, lower part of the cuneus and precuneus, inferior frontal and orbitofrontal cortices, the superior temporal cortex and the posterior part of the middle temporal cortex (Fig. 2b).

## Discussion

We presented two cohorts of patients with dystonic symptoms related to DBS. Both de novo dystonic patients and pre-dystonic patients who did not improve after DBS had a greater distance of active contact from STN and longer disease duration before surgery than other patients. No statistically significant differences were found in sex, body side first affected by PD, age at implantation, age at onset of PD and clinical scores before and after the implantation. Furthermore, antiparkinsonian medication dosage was not statistically different in our patients before and after the implantation. Our findings are in keeping with the worsening of dystonia with levodopa (‘peak dose dystonia’) already described in the literature [22]. Therefore, our findings seem to corroborate the hypothesis that dystonia is mainly caused by stimulation, particularly when delivered in discrete brain areas. Although cases of dystonia after STN-DBS exist in the literature, only focal dystonic symptoms – i.e., AEO and facial or limb cramps- were reported [23]. These were thought to originate from current spreading to internal capsule fibers, since they were usually responsive to voltage tapering [8]. The novelty of our work resides in the fact that we described the full spectrum of dystonic symptoms -both focal and generalized- after STN-DBS and that a subgroup of our patients displayed dystonic postures resistant to stimulation parameter changes.

The identified sour-spots extended medially from the STN towards the rZI. [17] While the sour-spot of de novo dystonic patients extended laterally to the fasciculus lenticularis, both sour-spots were in contact with the rZI. As a slit-like nucleus between STN and the thalamus, the ZI is divided in superior primates into caudal ZI (cZI) and rZI. cZI is closely connected to the basal ganglia and motor cortex and is probably responsible for the anti-dyskinetic and anti-tremor effects seen in ZI stimulation [24]. On the other hand, stimulation of the rZI seems to modulate a different network and has been involved in the pathogenesis of OCD and other neuropsychiatric disorders [25]. Since rZI receives afferents from widespread areas of the prefrontal cortex and sends efferents to structures connected to our sour-spot – namely, the cerebellum and the upper brainstem-, it appears reasonable to speculate a role of rZI in dystonia. The structural and functional connectivity profile of our sour-spots included areas known to be correlated with improved motor outcomes after STN-DBS in previous studies [17], i.e., the motor cortex, supplementary and pre-supplementary motor areas and the thalamus. However, our fingerprints also covered wider parts of other brain regions, which we can be distinguished into two groups: infratentorial structures (cerebellum and midbrain) and supratentorial areas (somatosensory and posterior parietal cortices, superior temporal gyrus).

The cerebellum is a common link between the thalamus and the basal ganglia because of a di-synaptic pathway connecting the dentate nucleus to the striatum via the thalamus and STN to the cerebellar cortex via pontine nuclei as demonstrated in primate tracing studies [26]. Evidence of cerebellar involvement in dystonia pathophysiology stems from animal studies (dystonic movements caused by lesioning of cerebellar nuclei in mice) [27] and neuroimaging research (altered cerebellar activity on functional MRI (fMRI) in sporadic and hereditary dystonia) [28]. Furthermore, clinical reports described the disappearance of dystonia after cerebellectomy [29] and the positive effect of cerebellar stimulation on treating dystonia [30]. Even deep nuclei in the upper brainstem were linked to clinical manifestations of dystonia. For instance, abnormal head movements mimicking cervical dystonia were evoked by ablation of the interstitial nucleus of Cajal (INC) [31]. This small nucleus is located in a plane between the midbrain and the diencephalon and oblique to the Forel’s H field and the periventricular grey matter in the subthalamic area. The INC coordinates vertical gaze responses to head movements along with other nuclei in the medial longitudinal fasciculus, as it receives strong afferents from the cerebellum via the superior cerebellar peduncle and the vestibular nuclei and sends efferents to the third and fourth cranial nerves and the cervical spinal cord. Although the role of the INC in cervical dystonia may be speculated due to its anatomical location and connections, INC stimulation was attempted in the past for the treatment of spasmodic torticollis, but its results were inconsistent [32]. Additionally, there are reports of pontomesencephalic lesions causing secondary dystonia, which subsided after lesion removal [33].

Along with the already discussed subcortical areas, several cortical areas resulted structurally and functionally connected to our sour-spots. In this context, it is worth noticing that a key distinctive feature of dystonia is the pathogenetic role of the cortex in the form of altered synaptic plasticity. Indeed, loss of sensorimotor inhibition was demonstrated in patients with focal dystonia using increased somatosensory temporal discrimination after high-frequency somatosensory stimulation [34]. Moreover, fMRI studies showed decreased or altered metabolism in the somatosensory and posterior parietal cortices and increased activation in the inferior frontal and temporal cortices. These abnormal activities in frontal and temporal areas were related to decreased surrounding inhibition from the somatosensory areas [35]. Interestingly, the same parietal and frontal areas connected to our sour-spots were negatively correlated with the clinical outcome in the aforementioned study by Horn et al. [19] and the altered activation in the somatosensory cortex was demonstrated to be reversible after GPi-DBS with optimized stimulation parameters [36]. In this context, a recent study [37] identified a dysfunctional network connecting STN with sensorimotor parietal and cerebellar cortices in dystonia patients, thus corroborating the results of our study. The functional fingerprint of the sour-spots in our study also extended to the anterior portion of the superior temporal gyrus, whose activity has already been negatively correlated to the clinical outcome of patients receiving GPi-DBS for both generalized and cervical dystonia [19]. On the other hand, other stimulation-related side effects were correlated to the over-activation of different brain areas than the ones described for dystonia [38]: the supplementary motor area for hyperkinesia and dysarthria, the frontal eye fields (Brodman area 8) for extraocular motility dysfunctions, the lemniscal fibers running through the posterior limb of the internal capsule for paresthesias.

Based on our results, we hypothesize a two-hit model for dystonia onset after STN-DBS, like what was proposed for secondary cervical dystonia from brain lesions by Corp et al. [39]. The first hit is a clinical feature of PD patients that can cause pre-existent altered plasticity on a cortical and subcortical level and predispose to the development of dystonic symptoms. We speculate that a longer disease duration before the implantation may be responsible for this state of predisposing dysfunctional plasticity. In fact, PD duration before the surgery was the only clinical feature displaying statistical significance in our analysis. This observation may be sustained by neuroimaging studies that observed altered function in frontal, temporal and parietal areas even in healthy carriers of genetic mutations of some hereditary forms of dystonia [28]. Both de novo dystonic and not-improved pre-dystonic patients were more frequently males and younger at PD onset, although none of these variables resulted statistically different from control patients. On the other hand, the second hit is the stimulation of an anatomical site connected with subcortical (cerebellum and upper brainstem) and cortical (prefrontal, parietal and temporal cortices) areas related to dystonia pathophysiology. All these anatomical areas are tightly compact crossroads of fibers, whose stimulation can easily overlap and activate further structures. According to this hypothesis, we speculate about two possible scenarios: non-dystonic PD patients with longer disease duration become dystonic after STN-DBS with stimulation spreading into “dystonicogenetic” areas; on the other hand, PD patients with dystonic symptoms do not improve after STN-DBS because the stimulation of the aforementioned areas perpetrates dystonia-related circuits and/or because stimulation wasn’t delivered in the most effective part of the STN. Taken together, this evidence reinforces the concept of dystonia as a “circuitopathy” [40], in which dystonic symptoms can result from combined dysfunction of some nodes or aberrant communication among the nodes in the circuit [26].

### Limitations

The main limitation of our work is its retrospective nature, which may lead to selection bias. Moreover, the dystonic patients displayed heterogeneous characteristics in dystonia type and temporal profile, even though the whole cohort was quite homogeneous for clinical variables (sex, body side first affected by PD, age at implantation, LEDD before surgery) and clinical outcome (ΔLEDD). For example, in keeping with the two-hit hypothesis our findings may explain why 56.2% of neo-dystonic patients improved after reducing the intensity of the stimulation (thus attenuating the effect of the second hit) but they do not account for the remaining neo-dystonic patients still not improving. Prospective studies with larger patient cohorts are needed to investigate whether variables that did not reach statistical significance or were not considered in our study may behave as first-hit. We based our sour-spot analysis on the N-image method to anatomically localize the side effects. Therefore, our two sour-spots represent the aggregates of VTAs for patients who developed dystonia after STN-DBS and continued to be dystonic even with the stimulation, respectively. Since we lacked quantitative measures of dystonia for each patient via clinical scales, we did not perform quantitative sour-spot analysis and calculate voxel-wise correlations of clinical scores with the derived fingerprints [41]. Furthermore, binarized VTA models are generated from applied thresholds and theoretical assumptions aiming to approximate the tissue stimulated by the surrounding axons. However, current VTA modeling is based on simplifying biophysical mechanisms behind DBS stimulation; hence, they do not afford indisputable neuroanatomical accuracy [42]. Finally, we used a Parkinsonian normative connectome for the fingerprint analysis. These data may not reflect patient-specific connectivity, even though the PPMI connectome we chose for this study is pathology-specific. However, normative connectomes offer superior data quality to patients’ native imaging performed with clinical scans, since normative data initiatives employ specialized hardware and optimized acquisition parameters with better spatial resolution, signal-to-noise ratio and fewer artifacts [43]. Although the hypothesis that the same fibers negatively associated with clinical outcomes in GPi-DBS for dystonia [19] would also be responsible for de novo dystonia after STN-DBS is intriguing, our results showed that other closer anatomical areas are covered by the sour-spots. Therefore, other components of the striato-thalamic and pallido-thalamic pathways or even different fibers may be involved.

## Conclusions

We analyzed a cohort of 60 Parkinsonian patients and then applied connectivity-based analysis to explain the development or persistence of dystonic symptoms after STN-DBS. Based on our results, we hypothesize a two-hit model of dystonia onset, according to which synaptic changes caused by longstanding PD act in synergy with the stimulation of dystonia-related brain areas.

Moreover, our work confirmed the concept of dystonia as an epiphenomenon of altered brain connection at multiple levels (basal ganglia, cerebellum, thalamus, midbrain and cerebral cortex). We are confident that this “connectomic” perspective will contribute to better understanding and treating this difficult and disabling disease.

## Supplementary Information

Below is the link to the electronic supplementary material. 

ESM1 (DOCX 499 KB)

## Data Availability

Data are available under reasonable request.
