# Supplementary material for: Exploring the relationship between dystonia and STN-DBS in Parkinson’s disease: insights from a single-centre cohort
Source: Neurol Sci. 2025 May 15;46(8):3691–701. doi: 10.1007/s10072-025-08230-7 (PMC12267303; doi:10.1007/s10072-025-08230-7)
Supplement: Supplementary file 1 — (DOCX 499 KB) [file 10072_2025_8230_MOESM1_ESM.docx]

**Supplementary Figure 1:** Histograms describing differences in gender among the four groups of patients.

**
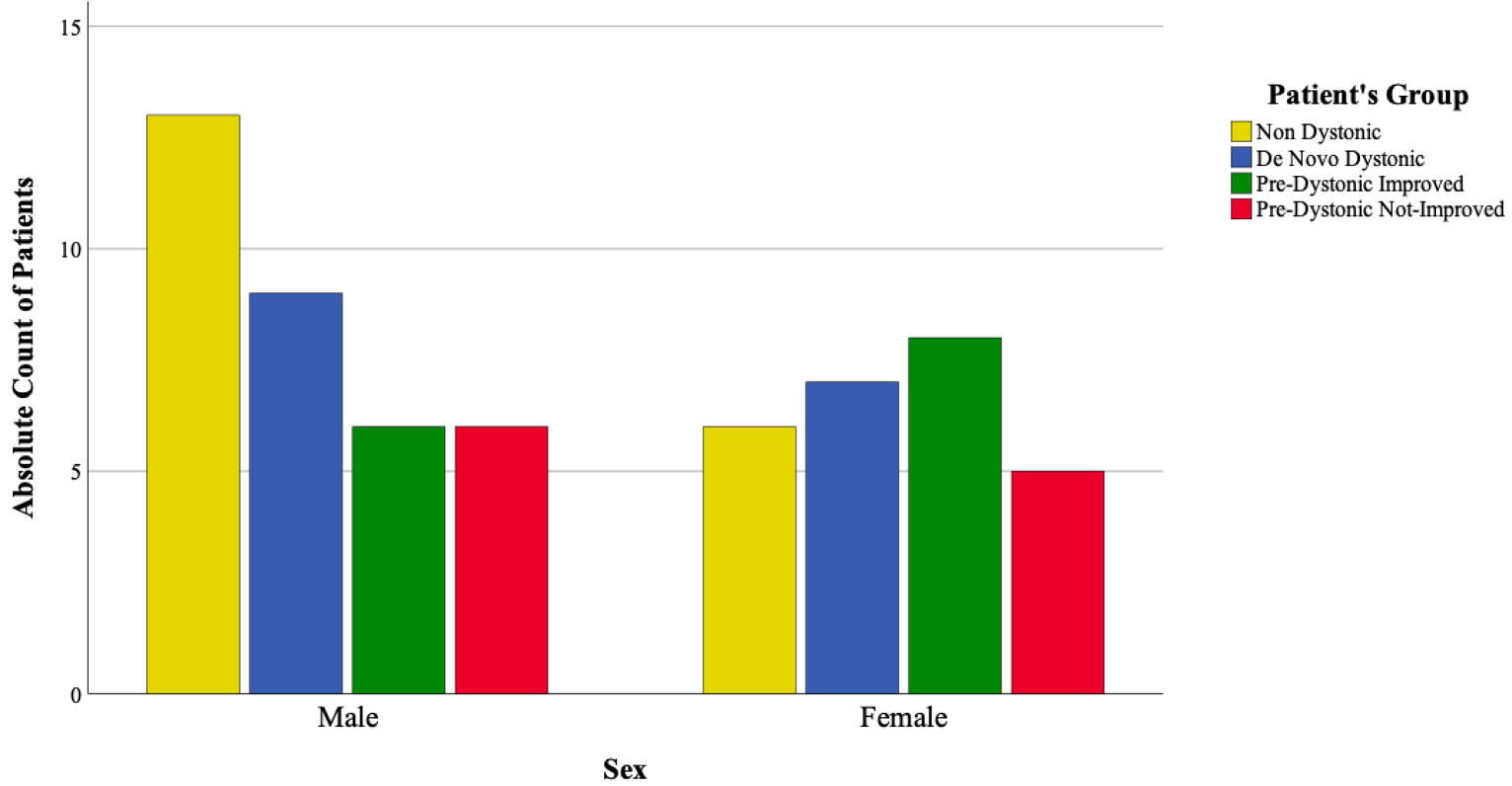
**

**Supplementary Figure 2:** Histograms describing differences in categorical variables among the three types of dystonic patients.

**
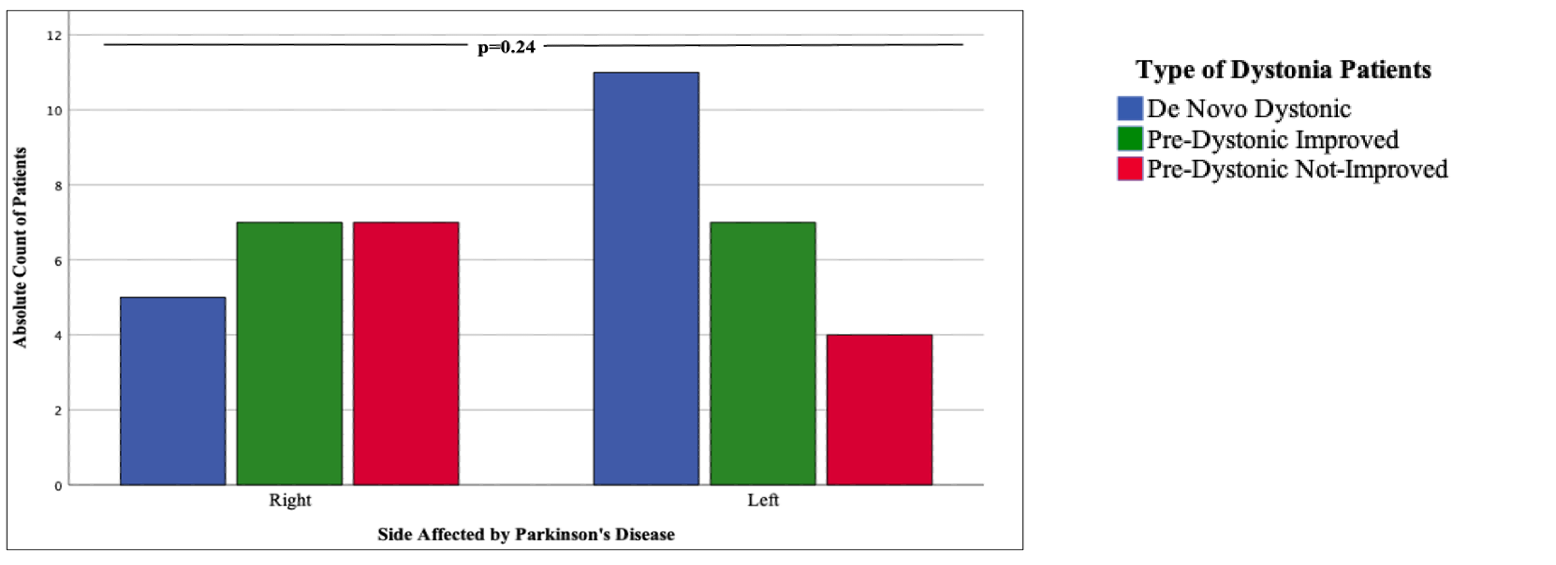
**

**Supplementary Table 1:** Results from the post-hoc tests.

| **Post-hoc Comparisons** | | **AC-STN^1^ Left** | **AC-STN^1^ Right** | **Disease duration^2^** |
| --- | --- | --- | --- | --- |
| **De novo dystonic** | Not dystonic | 0.004 | 0.002 | 0.032 |
|  | Pre-dystonic not-improved | 0.95 | 0.994 | 0.96 |
|  | Pre-dystonic improved | 0.015 | 0.003 | 0.04 |
| **Not dystonic** | De novo dystonic | 0.004 | 0.002 | 0.032 |
|  | Pre-dystonic not-improved | 0.037 | <0.001 | 0.002 |
|  | Pre-dystonic improved | 0.9 | 0.9 | 0.99 |
| **Pre-dystonic not-improved** | De novo dystonic | 0.95 | 0.994 | 0.96 |
|  | Not dystonic | 0.037 | <0.001 | 0.002 |
|  | Pre-dystonic improved | 0.01 | <0.001 | 0.04 |
| **Pre-dystonic improved** | De novo dystonic | 0.015 | 0.003 | 0.04 |
|  | Not dystonic | 0.9 | 0.9 | 0.99 |
|  | Pre-dystonic not-improved | 0.01 | <0.001 | 0.04 |

1: distance of the center of the active contact (AC) from the subthalamic nucleus (STN) in mm; 2: mean duration of Parkinson’s disease in years until the surgery.
